# Stock market optimization amidst the COVID-19 pandemic: Technical analysis, K-means algorithm, and mean-variance model (TAKMV) approach

**DOI:** 10.1016/j.heliyon.2023.e17577

**Published:** 2023-06-22

**Authors:** Maricar M. Navarro, Michael Nayat Young, Yogi Tri Prasetyo, Jonathan V. Taylar

**Affiliations:** a School of Industrial Engineering and Engineering Management, Mapúa University, 658 Muralla St., Intramuros, Manila 1002, Philippines; b School of Graduate Studies, Mapúa University, 658 Muralla St., Intramuros, Manila 1002, Philippines; c Department of Industrial Engineering, Technological Institute of the Philippines Quezon City, 938 Aurora Blvd, Cubao, Quezon City 1109, Metro Manila, Philippines; d International Bachelor Program in Engineering, Yuan Ze University, 135 Yuan-Tung Road, Chung-Li 32003, Taiwan; e Department of Industrial Engineering and Management, Yuan Ze University, 135 Yuan-Tung Road, Chung-Li, 32003, Taiwan; f Department of Computer Engineering, Technological Institute of the Philippines Quezon City, 938 Aurora Blvd, Cubao, Quezon City 1109, Metro Manila, Philippines

**Keywords:** Technical analysis, Machine learning, Optimization, Stock market, COVID-19

## Abstract

The Philippine stock market, just like most of its neighbors in the region, was seriously impacted by the global pandemic COVID-19. Investors remain hopeful while continuing to seek great ones in the damaged market. This paper developed a methodology for portfolio selection and optimization with the use of technical analysis, machine learning techniques, and portfolio optimization model. The combined methods of technical analysis, K-means clustering algorithm, and mean-variance portfolio optimization model will result in the development of the proposed TAKMV method. The study aims to integrate these three important analyses to identify portfolio investments. This paper uses the average annual risk and annual rate of return data for the years 2018 and 2020 to form the clusters and assessed the stocks that correspond to the investor's technical strategy such as Moving Average Convergence/Divergence (MACD) and Hybrid MACD with Arnaud Legoux Moving Average (ALMA). This paper solved the risk minimization problem on selected shares of the companies, based on the mean-variance portfolio optimization model. There are 230 and 239 companies for 2018 and 2020, respectively, listed in Philippine Stock Market, and all simulations were performed in MATLAB environment platform. Results showed that MACD strategy dominates the MACD-ALMA strategy in terms of the number of assets with a positive annual rate of return. The MACD works efficiently in the pre-COVID-19 condition while MACD-ALMA works efficiently during-COVID-19 condition, regardless of the number of assets with a positive annual rate of return. The results also show that the maximum expected portfolio return (*R*_*P*_) can be achieved using the MACD and MACD-ALMA in the pre-and during-COVID-19 conditions, respectively. The MACD-ALMA shows an advantage during high-risk market conditions and can also provide maximum *R*_*P*_. The performance of the TAKMV method was validated by applying its results and comparing it to the next year's historical price. The 2018 results were compared to 2019 data and the 2020 results were compared to 2021 data. For consistency, the comparison was applied to the same company per portfolio. Simulation results show that the MACD strategy is more effective compared to MACD-ALMA.

## 1 Introduction

The worldwide stock market was globally affected by the COVID-19 pandemic. In most countries, stock markets were negatively influenced by the spread of the COVID-19 disease [[1], [2], [3], [4], [5], [6], [7], [8], [9]]. The Philippine Stock Market, also known as the Philippine Stock Exchange (PSE), has been significantly impacted by the COVID-19 pandemic. As with many stock markets around the world, the PSE has experienced volatility and uncertainty as investors react to the pandemic.

In the early months of the pandemic, the PSE saw significant declines in stock prices as investors responded to concerns about the impact of the pandemic on the Philippine economy. The PSE Index, which tracks the performance of the largest companies listed on the exchange, fell to its lowest level in years.

Despite the challenges posed by the pandemic, some investors have continued to invest in the Philippine Stock Market. Some have seen the pandemic as an opportunity to invest in companies that have the potential to thrive in the new normal, such as those in the technology and healthcare sectors. Investors in the Philippines have also had to adapt to changes brought about by the pandemic, such as the shift to remote work and digital channels. This has presented new challenges for investors who may be used to more traditional methods of investing. Investors remain hopeful about the Philippine Stock Market, while retail investors continue to seek great ones in the damaged market. These are generally young, tech-savvy millennials with a lot of money to trade online. Many big stock brokerage companies had to temporarily block new account openings or invest in additional bandwidth to accommodate the rush of online trades as a result of their rapid flood into the market. Retail investors' increased engagement is a strong sign that the local stock market still has plenty of chances for those willing to look beyond short-term gains. With increased knowledge of the local market and increased investment maturity, these investors have boosted their interest in listed shares, which allows the economy to recover more quickly from the pandemic's effect. Despite certain setbacks, like the continued rise in COVID-19 cases, which prompted the government to revert to more restrictive regulations in Metro Manila and neighboring provinces, the Philippine Stock Exchange Index (PSEi) is expected to have a stronger year in 2023 than it did in 2020–2022. In addition, the vaccine roll-out boosted the investor's confidence.

In today's world of rapid technological progress, institutional and retail investors in the Philippines have a variety of choices, including online trading. These types of investors can benefit from the online trading platform, though they are not all the same, and there are several distinctions between institutional investors and non-institutional, or retail, investors. An organization or person that trades assets in huge amounts to be eligible for privileged dealing and less expensive fees is known as an institutional investor, they do not invest their funds; instead, they invest the funds of others on their behalf. A retail investor, on the other hand, is an individual or non-professional investor who buys and trades stocks through brokerage firms. They frequently invest in brokerage or retirement accounts for their benefit. There is a significant increase in retail investors who used online stock trading platforms in the Philippines during COVID-19. From a newbie investor who requires assistance in developing an investment strategy to a seasoned investor who can use an online trading platform to execute a strategy.

In the Philippines, online stock trading platforms are widely used, making investment extremely convenient and accessible to the majority of Filipinos. It became the new normal's trend that supports the rapid spread of online investing and trading whether it was on mobile or web-based platforms. These platforms were targeted by institutional and retail investors and are widely accessible and given mostly by stock brokerage firms in the Philippines. In addition, these platforms were useful in assessing the stock performance of the top gainers and top losers for each day of individual stock performance. Institutional and retail investors can easily check their stock portfolio on these online trading platforms. The record of transactions is available online, and investors can buy and sell at any time based on their preference without having to depend on the broker which has complete control over the stock portfolio.

A stock portfolio is a collection of equities in which investors and traders specifically invest intending to make a profit. To become a resilient investor, one should diversify his investments that span several sectors, for the reason that if one area suffers a setback, the investments in other industries aren't necessarily impacted. Diversification is an important part of building a portfolio, yet full diversification is impossible because investing in each company needs a significant amount of money. Apart from that, not all companies are worth investing in, which could be due to their illiquidity. As an institutional or retail investor, it is obvious to develop a successful strategy that will select stocks that will give higher returns and lower risk.

Philippine Stock Market has faced challenges during this time, there are also opportunities for investors who are willing to navigate the new normal and identify companies with the potential to succeed in the post-pandemic world. Broad market pullbacks such as the COVID-19 pandemic situation obviously can suffocate the entire portfolio without any good strategy employed. In this study, we proposed a combined method of Technical Analysis, K-means Algorithm, and Mean-Variance Model named as TAKMV method. The diversification of this study will help to protect an investor's portfolio from the systematic risk that could expose the portfolio to losses. The TAKMV method was introduced to help investors, traders, managers, and decision-makers in analyzing the stock market and was used to identify possible portfolio investments.

## 2 Literature review

Portfolio selection is the process of determining a mix of securities from a huge variety of choices. It is a method of allocating wealth to a group of assets to attain long-term objectives [10,11]. Its purpose is to maximize investment returns for investors. According to Markowitz [12], investors must choose between profit maximization and risk minimization. Risk minimization for a predetermined level of return or return maximization for a calculated risk level is two options available to investors. Markowitz calculated investment return as the expected value of securities' profits. The divergence from the expected value, according to Markowitz, is a risk. The Mean-Variance (MV) optimization method solves the portfolio problem by combining two basic indicators such as expected returns (represented by the mean return) and risk (measured by the volatility of the return). Understanding the behavior of financial asset prices and predicting its future behavior has always been a difficult task for practitioners. As a result, precisely anticipating price swings is critical for making gratifying and profitable investing decisions [13]. Portfolio selection has been extensively researched in a variety of fields, including quantitative and conventional finance, machine learning, and artificial intelligence [14]. Prior research has focused on financial factors on different Investment behavior [[15], [16], [17], [18], [19]].

Fundamental and technical analysis has traditionally been the two most extensively used ways of analyzing stock market data [20]. The concept of intrinsic value is used in fundamental analysis, which means that the present price is based on both qualitative and quantitative data. Fundamental analysis seeks to determine a company's fair market value by exploring all components of the firm, as well as the market, industry, domestic and large-scale environment. Whoever conducts the analysis should analyze the firm's overall performance and financial statements, as well as all recent corporate news. Investors should determine if the market correctly incorporated all relevant data into the stock price. The investor must analyze all aspects of financial statements, including profits, assets, revenues, and expenses, do a year-by-year comparison, compare to industry standards, observe specific tendencies in their behavior, and value the shares appropriately based on all of this. While recent studies proposed different algorithm models to analyze the performance of the selected portfolio [[21], [22], [23], [24], [25]] using Fundamental variables, it has many limitations and flaws in the search for future price forecasting, making it even more difficult to construct models that accurately describe stock variations. Because it relies on a deeper understanding of all aspects of a company and individual stocks, accumulating all of these qualities for a thorough company valuation can be time-consuming and expensive, making it impracticable in many cases, especially from a retail investor's perspective.

Technical analysis, on the other hand, seeks to recognize a pattern in data, such as price movements and past returns that may be used to predict future price movement for securities and the market as a whole [26]. It is a well-known method that uses stock prices or technical indicators as inputs. Based on technical analysis, the stock price has already represented all of the major underlying variables, it focuses only on the stock price and trading volume, both of which are recorded and displayed in various tables and graphs. Investors can learn about specific trend forms and regularities in price movement, trading volume, and interdependence by evaluating these graphs. The high and low prices for each trading period, as well as the opening and closing prices, are plotted for each trading period. Moving averages are frequently used as technical analysis indicators in related literature such as Simple Moving Average [[27], [28], [29], [30]]; Weighted Moving Average [31]; Exponential Moving Average [28,32]. Some studies used Moving Average Convergence/Divergence (MACD) as a technical indicator with machine learning applications [27,29]. Fundamental analysis examines stocks over a longer period than a technical analysis which takes place over a short period, such as days, weeks, or months [33]. In the literature, variables such as macroeconomic, financial, and technical indicators have been examined as the most significant ones influencing stock price movements [34]. In most prediction studies, technical indicators play an essential role in buy and sell signals for stocks and are widely applied as input variables. Prior studies used technical analysis to examine stock behaviors [35,36].

While past research has focused on portfolio selection based on fundamental analysis, technical analysis should also be considered when using machine learning to select a portfolio. Clustering is an unsupervised data mining approach for grouping things based on their similarities. It's used to examine a variety of datasets. K-means is one of the most commonly used unsupervised clustering algorithms. However, it is difficult to determine the value of the k parameter, which represents the number of clusters, one of the most commonly used methods for determining the number of clusters is the cluster validity index. Numerous internal and external validity indexes are used to find suitable cluster numbers based on the characteristics of datasets [37]. In the stock market, Cluster analysis helps to distinguish stocks with different characteristics. Prior Studies include K-means on selecting stocks index, particularly in Asia. In the study of [38], they consider the data preprocessing using trimmed k-means clustering for robust mean-variance portfolio selection. The optimum portfolio is formed by selecting the stock representation for each cluster using the Sharpe ratio in the Indonesian Stock Exchange. Another paper studied the Fuzzy Time Series Markov Chain model [39] and offered a new strategy that combines a combination of absolute differences and k-means clustering. Based on the data it made the interval more flexible and compact. The Taiwan Capitalization Weighted Stock Index (TAIEX) was employed as a benchmark in their study. In addition [40], investigated the use of the K-means clustering method in stock forecasting, with the K-means algorithm being improved by incorporating the artificial fish swarm algorithm (AFSA) which is named KAFSA. Closing price, price-earning ratio, earnings per share, and return on net assets were used to verify the prediction results. The findings revealed that there were considerable variances between A and B equities when divided by KAFSA, with B stocks showing significantly larger discrepancies than A stocks. Moreover [41], proposed a model that takes into account heterogeneity and looks for homogeneous groups of enterprises with high importance. The multiple kernel learning technique and K-means clustering which is used to forecast stock price changes and incorporate information from the target company and its homogenous cluster. The experiment was conducted utilizing three years of data from the Republic of Korea. The results reveal that in the vast mainstream of circumstances, the suggested strategy outperforms current methods in terms of predictability. The findings also suggest that the need for cluster analysis is dependent on the sector's heterogeneity and that as the heterogeneity develops, it is necessary to undertake cluster analysis with a bigger number of clusters. Furthermore, the work of [42] proposed an analytical approach for determining thresholds and migration within clusters using simulations with k-means clustering and homogeneous Markov chains. Quarterly financial data from a sample of 35 public organizations from July 1, 2006 to March 28, 2020 (companies listed on the stock exchanges of the United States, Mexico, Brazil, and Chile) was used. Furthermore [43], established a six-step portfolio selection methodology in the Mongolian stock exchange, taking into account the economic, legal, political, and corporate governance implications. They used the methodology to acquire stock price information from companies in the TOP-20 index. Using the k-means method, grouping of share return, and risk evaluations, hierarchical clusters were created based on the correlation matrix of share return. They identified the most efficient portfolio and solved the return maximization objective on selected companies based on Markowitz's mean-variance model. Lastly, the work of [44] suggested a new indicator such as the Simple Moving Average of Price Change Ratios (SMA-PCR-N). It's an adjusted version of the standard Simple Moving Average (SMA). They show how to establish a diversified stock portfolio using k-means clustering and SMA-PCR-N. For the fiscal years 2015–2017, data on around 300 stocks were acquired from the Stock Exchange of Thailand and utilized in trials to evaluate the performance of their proposed approach. In most cases, portfolios built using SMA-PCR-N outperformed portfolios built with SMA clusters, according to the findings.

Assessing the risk and returns of multiple firms can be difficult; grouping more than two hundred thirty equities in the Philippine stock market is nearly possible with the help of the clustering technique. Cluster analysis can help by aggregating returns and risk so the investor or trader can concentrate on each group rather than trying to make decisions based on individual stocks. This study focused on the PSE, which was founded in 1992. Using the Philippine Stock Exchange (PSE), portfolio selection, like any other decision-making problem, is influenced by a variety of factors, both directly and indirectly. In this regard, researchers, managers, investors, and practitioners have found it difficult to investigate, recognize, rank, and use criteria to analyze, select, and optimize portfolios. Therefore, this paper developed a methodology for portfolio selection and optimization with the use of technical analysis, machine learning techniques, and a portfolio optimization model. TAKMV methodology, a combined method of Technical Analysis, K-means clustering algorithm, and Mean-Variance portfolio optimization model was proposed in this paper. The study aims to integrate these three important analyses to come up with the best portfolio. This paper uses the average annual risk and an annual rate of return data for the years 2018 and 2020 to form the clusters and assessed the stocks that correspond to investors' technical strategies such as Moving Average Convergence/Divergence (MACD) and Hybrid MACD with Arnaud Legoux Moving Average (ALMA). In the empirical experiment, To select the efficient portfolio, this paper solved the risk minimization problem on selected shares of the companies, based on the mean-variance portfolio optimization model. There are 230 and 239 companies for 2018 and 2020, respectively, listed in Philippine Stock Market, and all simulations were performed in MATLAB environment platform. To the best of the authors' knowledge, this is the first study to use this TAKMV method in the Philippine Stock Market pre- and during COVID-19 conditions. This paper can help to improve people's understanding of machine learning and technical analysis, as well as dispel their misconceptions about the difficulty of analyzing the stock market in the Philippines.

## 3 Research methodology

**Fig. 1:**
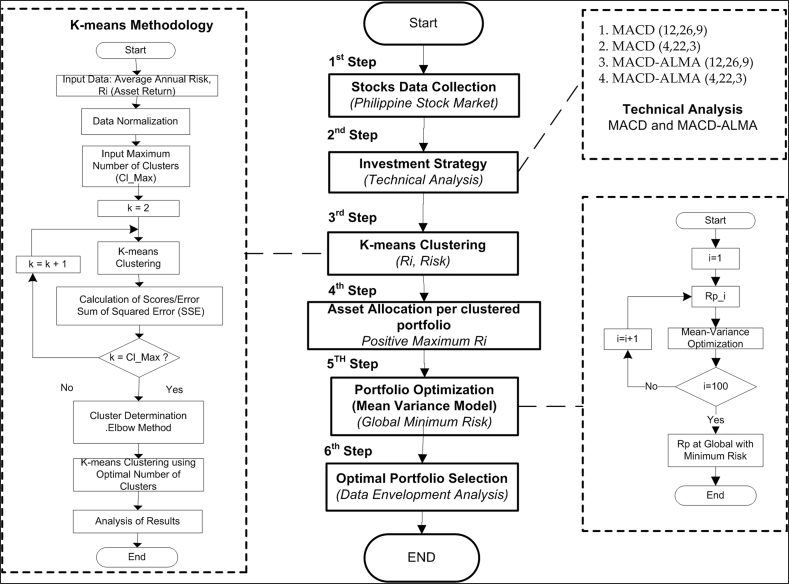
Flowchart of the proposed **TAKMV** methodology.

This paper consisted of five stages. Stage 1 constituted the data collection from Marketwatch [45]. Stage 2 was based on the investment strategy using a hybrid technical indicator: Moving Average Convergence/Divergence (MACD) and Arnaud Legoux Moving Average (ALMA). Stage 3 was stock clustering in the Philippine Stock Market based on average annual risk and an annual rate of return. Stage 4 evaluates stock average annual risks and returns according to clustered MACD or MACD-ALMA strategy. And lastly, Stage 5 identified the most efficient portfolio using the mean-variance portfolio optimization model. Combining three important analyses (**T**echnical **A**nalysis, **K**-Means Clustering, and **M**ean-**V**ariance Model) will result in the proposed **TAKMV** methodology. Fig. 1 shows the flowchart of the proposed methodology.

### 3.1 Stage 1: data collection

The data used in this study corresponded to the 2018 and 2020 historical prices of the Philippine Stock Market pre- and during-COVID-19 conditions. It composes of the Bank and Financial sector, Commercial and Industrial, Conglomerates, Consumer, Index, Insurance, mining and oil, Properties, Services, and Telecoms sectors with a total of 230 and 239 companies in the Philippines for 2018 (pre-COVID-19) and 2020 (during-COVID-19), respectively. To determine the performance of the model, it was then validated by applying the results and comparing it to 2019 (for 2018 results) and 2021 (for 2020 results) data. No validation was conducted between 2019 and 2020 since this was the transition of pre- and during-COVID-19 conditions.

### 3.2 Stage: 2: Technical analysis

Technical Analysis indicators are linear functions that calculate recurrent values using historical trading data such as open, high, low, or close prices, volume, open interest, advances, declines, and so on. Short-term investment benefited from the use of Technical Analysis and Price Actions. In this paper, we suggested a technical indicator for an investment strategy to identify profitable stocks, such as the Moving Average Convergence/Divergence Method (MACD) and Arnaud Legoux Moving Average (ALMA). The MACD (12, 26, 9), MACD (4,22,3), MACD-ALMA (12, 26, 9), and MACD-ALMA (4,22,3) were the technical investment strategies used in this paper.

#### 3.2.1 Moving average convergence/divergence (MACD) method

MACD stands for moving average convergence/divergence and is one of the most widely utilized momentum indicators in technical analysis. Gerald Appel created this at the end of 1970 [46]. It is usually used both for long-term and short-term investors. The MACD line is calculated as the difference between the 12th and 26th-day exponential moving averages. It explains that the MACD line and the MACD moving average line show oscillations around the zero-level line over time, as well as divergent, convergent, and crossover movements [47]. MACD is commonly used with *n*_*1*_, *n*_*2*_, and *n*_*3*_ combinations but other values can be substituted depending on goals. This is usually represented in the form MACD (*n*_*1*_, *n*_*2*_, *n*_*3*_). For the case of (12, 26, 9), it was represented as MACD (12, 26, 9). The MACD indicator was used in its classic form, which calculates using exponential moving averages and prioritizes the most recent data in its weighting process. It is calculated by subtracting the shorter exponential moving average (EMA) of window length *n*_*1*_ from the longer EMA of window length *n*_*2*_.

The MACD trading indicator consists of the following three elements.

1. The MACD line (equation (2)): the difference between the short- and long-term exponential moving average (EMA).
2. The Signal line (equation (3)): an exponential moving average of the MACD line.
3. Histogram (equation (4)): a graphical representation of the distance between the MACD line and the Signal line.

The EMA is defined in equation (1) as

(1) $$EMA_{t}\left(N\right)=\left[\frac{2}{N}\left(I_{t}-EMA_{t-1}\left(N\right)\right)\right]+EMA_{t-1}\left(N\right)$$

(2) $$MACD_{t}\left(n_{1},n_{2}\right)=EMA_{t}\left(n_{1}\right)-EMA_{t}\left(n_{2}\right)$$

(3) $$Signal_{t}\left(n_{3}\right)=\left[\frac{2}{n_{3}+1}\times \left(MACD_{t}\left(n_{1},n_{2}\right)-Signal_{t-1}\left(n_{3}\right)\right)\right]+Signal_{t-1}\left(n_{3}\right)$$

(4) $$Histogram_{t}=MACD_{t}-Signal_{t}$$

Where.

$EMA_{t}\left(N\right)$ = Exponential moving average at time *t*
*N* = window length of EMA (e.g., *n*_*1*_ and *n*_*2*_)
*n*_*3*_ = denotes the period for the EMA calculation of the MACD_*t*_ series
*I*_*t*_ = closing day price (index value) at time *t*
*Signal*_*t*_*(n3) =* EMA of MACD line at time *t*; (Signal Line)
*Signal*_*t-1*_ = initial previous signal line that starts at 2nd period EMA of MACD line at time t

#### 3.2.2 Moving average convergence/divergence (MACD) trading rules

The MACD and the signal line move up and down the zero axis or midline to show the following trends such as overbought or oversold. When the EMA points are close together, it is called convergence, and when they are far apart, it is called divergence. The MACD line reacts more strongly when the moving average is shorter. Signal line crossovers, centerline crossovers, and divergence are some MACD indicators. The signal line is the MACD line's EMA. As a result, it follows the average line and aids in detecting turns in the MACD. When the MACD crosses over the signal line, it shows bullish and is called a bullish crossover. A bearish crossover occurs when the price falls below the signal line. Below are the MACD trading rules used in this paper and is shown in equations (5), (6):

(5) $$Buy\;Signal:Histogram_{t}=MACD_{t}-Signal_{t}>0$$

(6) $$Sell\;Signal:Histogram_{t}=MACD_{t}-Signal_{t}<0$$

Also, the annual return is calculated as shown in equation (7),

(7) $$R_{A}=\sum_{i=1}^{M}P_{Sell}-\sum_{j=1}^{N}P_{Buy}$$

And the annual rate of return as shown in equation (8),

(8) $$R_{i}=\frac{R_{A}}{R_{BS}}\times 100\%$$

Where;
R_A_ = Annual Return
Ri = Annual Rate of Return
R_BS_ = Closing index value where the first transaction occurs (Buy or Sell)
M = number of sell signal
N = number of a buy signal
P_Buy_ and P_Sell_ are the closing index values on the days to execute buying and selling transactions, respectively

A positive (negative) value of *R*_*A*_ indicates a profit (loss), which is applicable in both the case of a long and a short trade. This paper uses the single-line crossover as a MACD indicator. Both MACD (12, 26, 9) and MACD (4,22,3) [48] were used in the analysis.

**Fig. 2:**
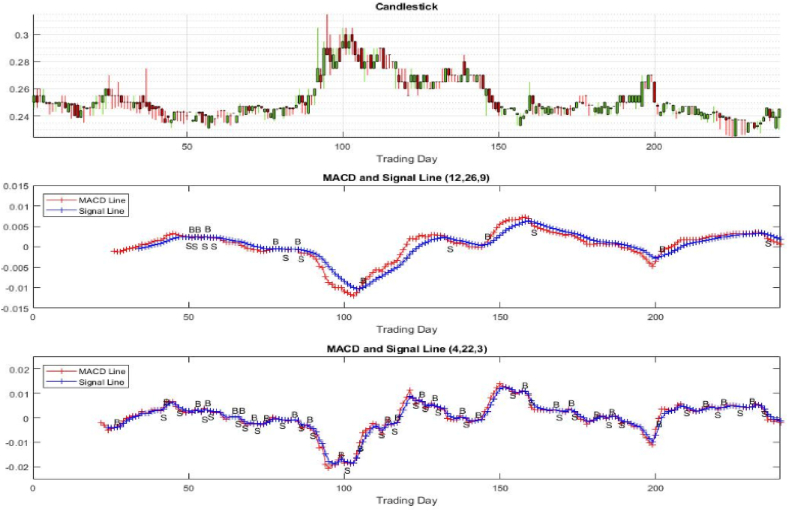
Trading rules for MACD (12, 26, 9) and MACD (4,22,3). (Upper: Candlestick; Middle: MACD and Signal Line of (12, 26, 9) Window; Lower: MACD and Signal Line of (4,22,3) Window).

Fig. 2 shows the same sample stock with the corresponding buying and selling index values for MACD (12, 26, 9) and MACD (4,22,3). “B” and “S” denote buying and selling point, respectively.

#### 3.2.3 Arnaud Legoux moving average (ALMA)

The Arnaud Legoux Moving Average, abbreviated ALMA, is a relatively new addition to the family of moving average technical indicators. The ALMA was created in 2009 by Arnaud Legoux and Dimitrios Kouzis Loukas and has quickly received attention in the trading community. It is a kind of weighted moving average, and the shape of the coefficient is a Gaussian filter. A normal Gaussian filter is a symmetrical bell type with the highest center, but ALMA uses an asymmetric Gaussian filter with the peak shifted to the nearest position to improve price tracking [49]. Because the ALMA is based on the moving average indicator, it is universally acceptable across markets and time frames. Equations 9–12 shows the calculation of ALMA line.

(9) $$ALMA=\frac{\sum \left(P_{i}C_{i}\right)}{\sum \left(C_{i}\right)},\frac{1}{Norm}\sum_{i=1}^{size}P\left(i\right)e\frac{{\left(i-offset\right)}^{2}}{\sigma^{2}}$$

(10) $$C_{i}=\exp\exp\left\{-\frac{{\left(i-offset\right)}^{2}}{\sigma^{2}}\right\}$$

(11) $$\sigma =\frac{N}{A}$$

(12) Offset=Truncation{B(N−1)}

Where.

*P*_*i*_ (*i* = 1,2,3, …,N) = is each closing price
*C*_*i*_ (*i* = 1,2,3, …,N) = is an arbitrary coefficient related to each closing price
*A*, *B*, *N* are arbitrary, and (*A* = 6, *B* = 0.85 is the default)

ALMA's formula is shown in (9), and it employs a Gaussian distribution shift with an offset so that it is not evenly centered on the window but is biased towards the more recent days. The offset can be adjusted, allowing us to trade off smoothness and responsiveness. The second parameter is the sigma parameter, which alters the shape of the filter, making it wider (larger sigma) or more focused (smaller sigma) (smaller sigma). The default value of 6 was inspired by the six sigma process, which provides excellent performance.

#### 3.2.4 MACD-ALMA trading rules

For this paper, ALMA is integrated into the MACD trading rules. If the signal and MACD were bullishly crossed in a buying condition, the investor would buy stocks. It signals a buying position if MACD is in an upward trend and the closing or opening price is higher than ALMA. The Sell position, on the other hand, will state that if the opening and closing price were both below ALMA, the first selling point should be sold immediately. This two-indicator hybrid was employed in a clustered group to select stocks that match the investing strategy requirements of MACD and ALMA. Below are the MACD-ALMA trading rules used in this paper and is shown in equations (13), (14):

(13) $$Buy\;Signal:Histogram_{t}=MACD_{t}-Signal_{t}>0\;and\;ALMA_{t}<P_{\left(O,C\right)t}$$

(14) $$Sell\;Signal:First\;ALMA_{t+m}>P_{\left(O,C\right)t}\;after\;Buy_{t}$$

Where.

(*t* + *m*) is the point after *Buy*_*t*_
$P_{\left(O,C\right)t}$ are opening and closing points

The calculation of MACD, signal line, histogram, annual return, and the annual rate of return is the same as in MACD. MACD-ALMA (12, 26, 9) and MACD-ALMA (4,22,3) were also examined.

**Fig. 3:**
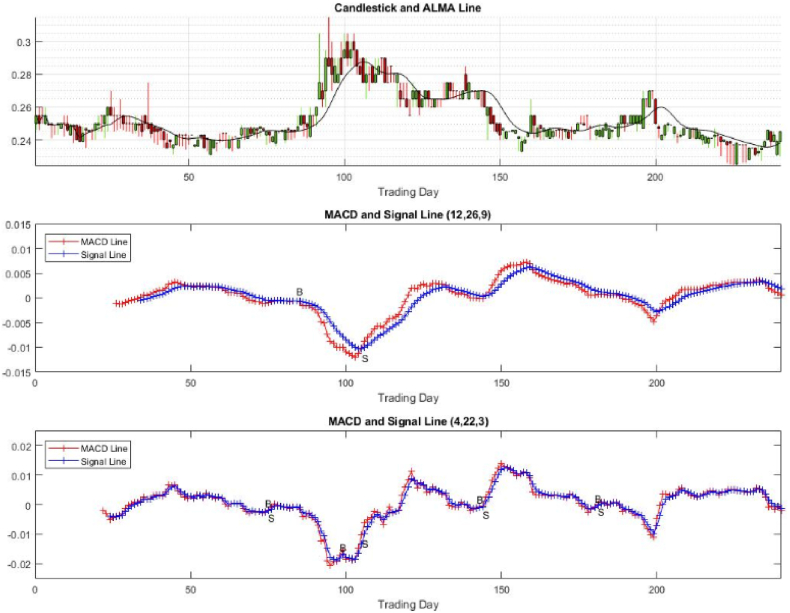
Trading rules for MACD-ALMA (12, 26, 9) and MACD-ALMA (4,22,3) (Upper: Candlestick and ALMA Line; Middle: MACD and Signal Line of (12, 26, 9) Window; Lower: MACD and Signal Line of (4,22,3) Window).

Fig. 3 shows the same sample stock with the corresponding buying and selling index values for MACD-ALMA (12, 26, 9) and MACD-ALMA (4,22,3).

### 3.3 Stage: 3: K-means algorithm

K-means Algorithm was used to cluster the Philippine Stock Market. The input data used to evaluate clusters was the annual rate of return and average annual risk. The greatest number of possible clusters investigated in this study was 20. The proposed elbow method was utilized to find the best cluster. K-means algorithm was also used to determine the cluster centroid and cluster labels for each data set. Likewise, the cluster profile or characteristics were investigated and analyzed.

Unsupervised learning models include the K-means clustering algorithm. Unsupervised models are used to learn from unlabeled or uncategorized data [50]. It searches for commonalities in the data set and responds to the presence or absence of such commonalities in each data point. A K-means clustering model starts with K centroids and categorizes data points that are close (similar) to the centroids as clusters [51]. Below is the step-by-step procedure of the K-means clustering algorithm.

Input: clusters number.

1st *Step*: (Initialization): Generate initial centroid.

*2nd Step*: (Assignation): Data point assignment to the nearest cluster (the nearest centroid).

*3rd Step*: (Re-computation): Update centroid.

4th *Step*: Calculate differences between the old and new centroid of each cluster. If the difference is lower than the tolerance limit, then stop. Otherwise, return to the 2nd Step.

#### 3.3.1 Input data: average annual risk and annual rate of return

In financial terms, risk is defined as the possibility that the real profits from an outcome or investment will differ from the expected outcome or return. The danger of losing some or all of the initial investment is a risk. While it is true that stocks are the most volatile of all investments, investors should keep in mind that uncertainty is an inherent part of the investing process. This means that any investment entails some level of risk. Limiting and managing your risk would be a healthier approach. A maximum level of income or loss should be established, and when that level is achieved, calculated decisions should be taken. Additionally, an investor's equivalent annual return over a specific time period is used to compute an annualized rate of return. Equations 15–19 shows the calcuation of daily return, variance of annual daily return, annual return, standard deviation of annual daily return, and annual rate of return, respectively.

(15) $$DR_{t}=\frac{P_{t}-P_{t-1}}{P_{t-1}}\times 100\%$$

(16) $${\sigma_{DR}}^{2}=\frac{\sum_{i=1}^{n}{\left({DR}_{t}-{DR}_{Ave}\right)}^{2}}{n-1}$$

(17) $$R_{A}=\sum_{i=1}^{M}P_{Sell}-\sum_{j=1}^{N}P_{Buy}$$

(18) $$\sigma_{DR}=\sqrt{\sigma^{2}}$$

(19) $$R_{i}=\frac{R_{A}}{R_{BS}}\times 100\%$$

Where.

*DR*_*t*_ = Daily return at *t* time period
*P*_*t*_ = Stock Closing Price at actual *t* time period
*P*_*t-1*_ = Stock Closing Price at previous *t* time period
*DR*_*Ave*_ = annual average daily return
*n* = no. Of daily returns in a year
*σ*_*DR*_^*2*^ = variance of annual daily return
*σ*_*DR*_ = standard deviation of annual daily return
*σ* = standard deviation of stock per year
*R*_*A*_ = Annual Return
*Ri* = Annual Rate of Return
*R*_*BS*_ = Closing index value where the first transaction occurs (Buy or Sell)
*M* = number of sell signal
*N* = number of a buy signal

**Fig. 4:**
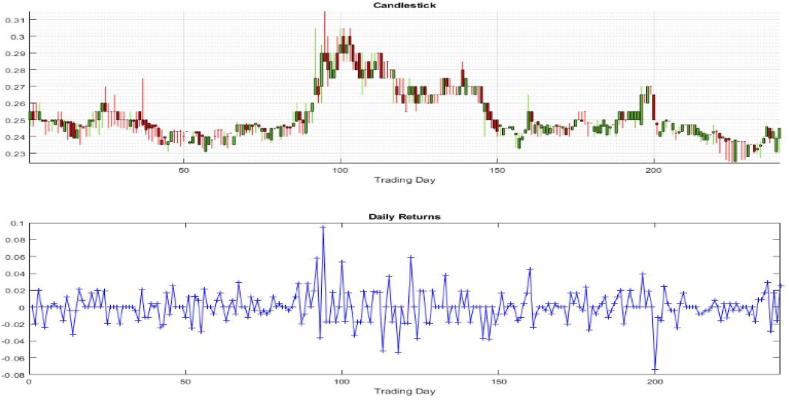
Sample stock data and daily return (Upper: Candlestick; Lower: Daily Return).

*P*_*Buy*_ and *P*_*Sell*_ are the closing index values on the days to execute buying and selling transactions, respectively. Fig. 4 shows an example of stock data plotted in candlestick form and corresponding daily returns for the entire year.

#### 3.3.2 Min-max normalization

In this paper, min-max normalization was used. The minimum value of each feature is transformed to a 0 value, the highest value is transformed to a 1, and the remaining values are converted to a decimal between 0 and 1. Normalizing the data so that they are all on the same scale means that each attribute is properly weighed before being used in the model [52]. The highest and smallest numerical values of each numeric value are determined, and the others are transformed correspondingly as shown in equation (20).

(20) $$Y^{\prime }=\frac{y+y}{y-y}$$

Where.

*Y’* = transformed value/pre-processed value
*y* = observation value
y _min_ = minimum observation value, and
y _max_ = maximum observation value.

The values in the dataset are reduced to {0, 1}.

#### 3.3.3 Elbow method

The elbow approach uses the sum of squared distance (SSE) to find an optimum value of *k* depending on the distance between data points and their allocated clusters [53]. We pick a *k* number where the SSE starts to flatten out and an inflection point appears. The method's name comes from the fact that this graph resembles an elbow when displayed. The goal is to have a low SSE *k* value. This paper uses the “knee locator” function for the elbow method to exactly determine the optimal number of clusters.

### 3.4 Stage: 4: evaluation of stock rate of return per cluster (asset allocation)

After calculating the rate of return (*Ri*) using the trading rules discussed previously, the next thing is to select the possible number of companies per cluster to include in the portfolio. This paper set some rules for portfolio selection.

1. Eliminate companies with a negative *Ri*
2. Eliminate companies with a window below MACD or ALMA window
3. Up to 10 companies per cluster. 10 is just an arbitrary number to simplify the analysis. Though it can be less than or more than 10. Based on author's own perspective and experience, 10 or more companies are difficult to manage and monitor.
4. Select companies with the highest *Ri* per cluster

### 3.5 Stage 5: portfolio optimization (mean-variance model)

According to the mean-variance model, investors prefer the security with the higher return considering specific risks, or the one with the lower risk based on a specific expected return. It is accomplished by evaluating the amount of risk that investors are prepared to take in return for rewards [54]. Since one of the clustering attributes used was an average annual risk, this paper uses a lower risk based on a specific expected return criterion. Equation (21) shows the objective function while equations (22), (23) are the contraints.

The model is formulated as a minimization problem:

(21) $$\mathrm{Min}\;\left({\sigma_{P}}^{2}\right)=\sum_{i=1}^{n}{\sigma_{i}}^{2}{w_{i}}^{2}+2\sum_{i=1}^{n-1}\sum_{j=i+1}^{n}w_{i}w_{j}\sigma_{ij}\rho_{ij}$$

Subject to:

(22) $$R_{P}=\sum_{i=1}^{n}w_{i}R_{i}$$

(23) $$\sum_{i=1}^{n}w_{i}=1$$

Where.

*σ*_*p*_^*2*^ = denotes the variance of portfolio *P*
*w*_*i*_ = denotes the weight on asset *i*
*σ*_*i*_ = denotes the standard deviation of asset *i*
*σ*_*ij*_ = denotes the covariance of asset *i* and asset *j*
$\rho_{ij}$ = denotes the correlation between asset *i* and asset *j*
*R*_*P*_ = Expected portfolio return
*Ri* = Annual Rate of Return

### 3.6 TAKMV simulation method

2018 (pre-COVID-19) and 2020 (during-COVID-19) data with 230 and 239 companies, respectively, were used to calculate each company's annual rate of return (*Ri*) based on the proposed technical investment strategies (MACD and MACD-ALMA) and trading rules. *Ri* may be a positive or negative value that indicates gain or loss or a “zero” value that indicates that the stock window is below the required window or there is no buying/selling point. Only the companies with positive *Ri* will be considered in the entire analysis. In parallel, average annual risks were also computed for each company. This resulted in an average annual risk and an annual rate of return per company per strategy. The average annual risk and an annual rate of return will be the attributes of the K-means clustering algorithm. The elbow Method was used to determine the optimal number of clusters. The resulting clusters will be the portfolio used in the optimization. To simplify the analysis, each portfolio was limited to 10 companies with the highest *Ri*. Each portfolio was simulated based on a mean-variance model that uses a lower risk based on a specific expected return criterion.

## 4 Results and discussion

The data used were composed of 230 and 239 companies in the Philippines for 2018 (pre-COVID-19) and 2020 (during-COVID-19), respectively. This was then validated by applying the results and comparing them to 2019 and 2021 data, respectively. All simulations in the proposed method such as technical analysis, K-means clustering, and portfolio optimization were all conducted in the Matlab platform.

### 4.1 Technical analysis results

**Fig. 5:**
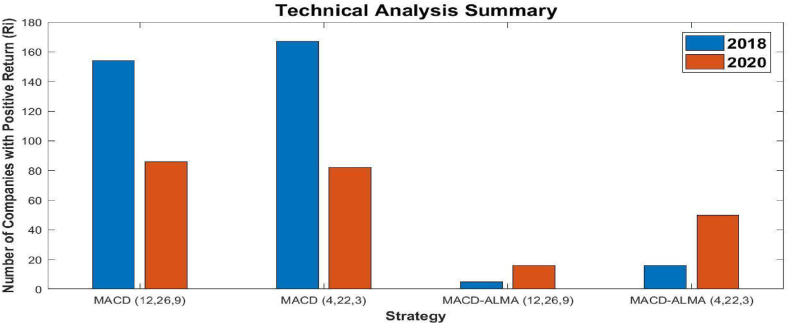
No. Of companies with positive *Ri* in different technical investment strategies.

The MACD (12, 26, 9), MACD (4,22,3), MACD-ALMA (12, 26, 9), and MACD-ALMA (4,22,3) were used to determine the annual rate of return (*Ri*) of individual companies per cluster group and highly dependent on the trading rules. The annual rate of return (*Ri*) may be a positive (negative) value that indicates gain (loss) or a “zero” value that indicates that the stock window is below the required MACD window or there is no buying/selling point. Only the companies with positive *Ri* will be considered in the entire analysis. Table 1 shows the summary of results for 2018 and 2020 data, respectively. Fig. 5 shows the graphical comparison of the number of companies with positive *Ri* for 2018 and 2020 data.

**Table 1:** Technical analysis summary of 2018 and 2020 data.

Technical Investment Strategy	2018 Data (230 Companies)			2020 Data (239 Companies)		
	+*Ri*	-*Ri*	“0″ *Ri*	+*Ri*	-*Ri*	“0″ *Ri*
MACD (12,26,9)	154	71	5	86	146	7
MACD (4,22,3)	167	62	1	82	157	0
MACD-ALMA (12,26,9)	5	76	149	16	95	128
MACD-ALMA (4,22,3)	16	108	106	50	101	88

Based on Table 1, for 2018 Data, (pre-COVID-19) MACD strategy dominates the MACD-ALMA strategy regarding the number of companies with positive *Ri*. MACD (12, 26, 9) generates 66.96% (154/230) number of companies with positive *Ri*, while MACD (4,22,3), generates 72.61% (167/230) positive *Ri*. Meanwhile,

During-COVID-19 condition (2020 data), the MACD strategy still dominates the MACD-ALMA regarding the number of companies with positive *Ri*. MACD (12, 26, 9) generates 35.98% (86/239). Of companies with positive *Ri*, while. MACD (4,22,3), generates 34.31% (82/239) number of companies with positive *Ri*.

The MACD Strategy greatly affects its performance during COVID-19 conditions and receives a huge reduction of companies with positive *Ri*. From 66.96% down to 35.98%. For MACD (12, 26, 9), while 72.61% down to 34.31% number of companies with positive *Ri* for MACD (4,22,3). This only shows how the pandemic hit the stock market with regard to the effectiveness of the MACD strategy.

However, the MACD-ALMA (12, 26, 9) and MACD-ALMA (4,22,3) generates 2.17% (5/230) and 6.96% (16/230) number of companies with positive *Ri* during Pre-Covid 19 (2018 data). During COVID-19 conditions (2020 data). , Both windows in MACD-ALMA Strategies surprisingly increase their performance. MACD-ALMA (12, 26, 9) generates 6.69% (16/239). Percent number of companies with positive *Ri*, while. MACD-ALMA (4,22,3) generates a 20.92% (50/239) number of companies with positive *Ri*.

MACD (12, 26, 9) is superior compared to MACD (4,22,3) regarding the number of companies with positive *Ri*. MACD-ALMA (4,22,3) is superior compared to MACD-ALMA (12, 26, 9) regarding the number of companies with positive *Ri*.

### 4.2 K-means clustering results

The 2018 and 2020 data were run in the K-means algorithm taking the average annual risk (σ) and an annual rate of return (*Ri*) as the clustering attribute. The Elbow Method was used to determine the best clusters for the given data. The cluster centroids would be the basis of the level of risk per cluster.

For the 2018 data (230 companies), the simulation shows that the best clusters were 8, 8, 2, and 4 for MACD (12, 26, 9), MACD (4,22,3), MACD-ALMA (12, 26, 9), and MACD-ALMA (4,22,3), respectively. For 2020 data (239 companies), the best clusters were 7, 7, 4, and 6 for MACD (12, 26, 9), MACD (4,22,3), MACD-ALMA (12, 26, 9), and MACD-ALMA (4,22,3), respectively.

**Fig. 6:**
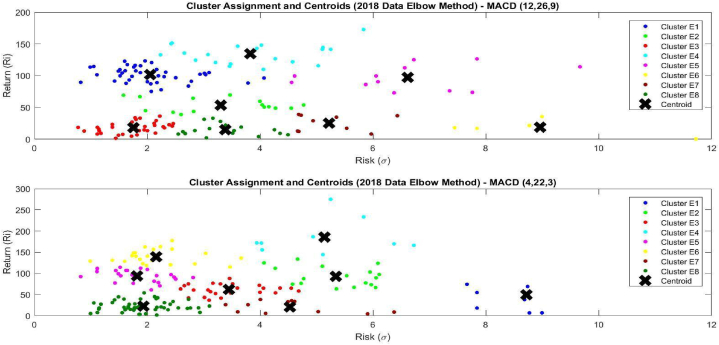
Cluster assignments and centroid for 2018 data (upper: Macd (12, 26, 9); lower: Macd (4,22,3)).

**Fig. 7:**
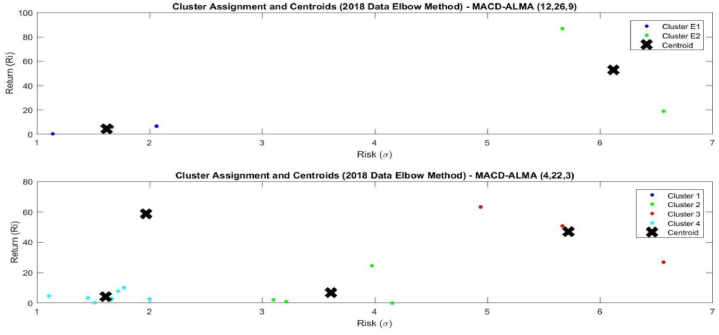
Cluster assignments and centroid for 2018 data (upper: MACD-ALMA (12, 26, 9); lower: MACD-ALMA (4,22,3)).

**Fig. 8:**
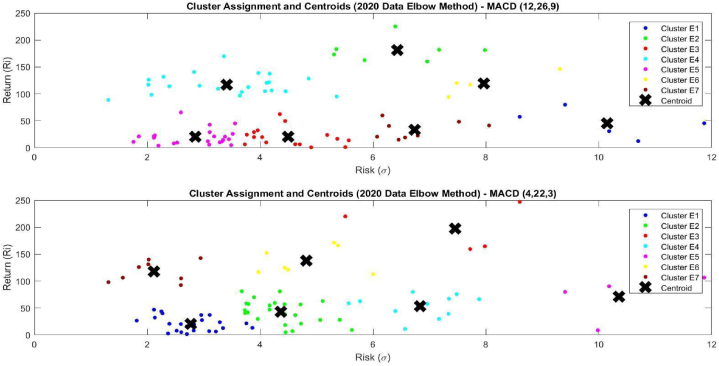
Cluster assignments and centroid for 2020 data (upper: Macd (12, 26, 9); lower: Macd (4,22,3)).

**Fig. 9:**
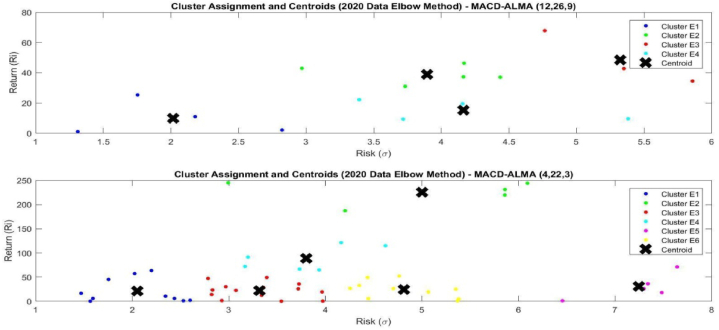
Cluster assignments and centroid for 2020 data (upper: MACD-ALMA (12, 26, 9); lower: MACD-ALMA (4,22,3)).

Table 2, Table 3 show the summarized results of the Elbow Method for 2018 and 2020, respectively. Fig. 6, Fig. 7 shows the graphical representation of 2018 cluster assignment and centroid for MACD and MACD-ALMA, respectively, while Fig. 8, Fig. 9 shows the graphical representation of 2020 cluster assignment and centroid for MACD and MACD-ALMA, respectively.

**Table 2:** Cluster centroid for 2018 data.

MACD (12,26,9)				MACD (4,22,3)				MACD-ALMA (12,26,9)				MACD-ALMA (4,22,3)			
Cluster	N + _*Ri*_	σ_(C)_	*Ri*_(C)_	Cluster	N + _*Ri*_	σ_(C)_	*Ri*_(C)_	Cluster	N + _*Ri*_	σ_(C)_	*Ri*_(C)_	Cluster	N + _*Ri*_	σ_(C)_	*Ri*_(C)_
E1	40	2.05	101.70	E1	8	8.72	49.48	E1	3	1.62	4.27	E1*	1	1.97	58.86
E2	15	3.30	53.96	E2	17	5.34	93.19	E2	2	6.12	52.88	E2	4	3.61	6.90
E3	36	1.75	18.22	E3	25	3.45	61.87	–	–	–	–	E3	3	5.72	46.98
E4	20	3.83	134.77	E4	9	5.14	186.10	–	–	–	–	E4	8	1.61	4.26
E5	12	6.62	97.27	E5	26	1.82	93.66	–	–	–	–	–	–	–	–
E6	5	8.96	18.69	E6	22	2.16	139.76	–	–	–	–	–	–	–	–
E7	9	5.22	25.44	E7	11	4.53	21.07	–	–	–	–	–	–	–	–
E8	17	3.38	14.99	E8	49	1.93	22.64	–	–	–	–	–	–	–	–

Where: N + _*Ri*_ - number of companies with positive *Ri*.

σ_(C)_ - Risk per Clustered Portfolio.

*Ri*_*(*C)_ - Clustered Portfolio Return.

Note: *E1 of MACD-ALMA (4,22,3) should be removed since only 1 company in a cluster.

**Table 3:** Cluster centroid for 2020 data.

MACD (12,26,9)				MACD (4,22,3)				MACD-ALMA (12,26,9)				MACD-ALMA (4,22,3)			
Cluster	N + _*Ri*_	σ_(C)_	*Ri*_(C)_	Cluster	N + _*Ri*_	σ_(C)_	*Ri*_(C)_	Cluster	N + _*Ri*_	σ_(C)_	*Ri*_(C)_	Cluster	N + _*Ri*_	σ_(C)_	*Ri*_(C)_
E1	5	10.15	45.22	E1	23	2.77	20.76	E1	4	2.02	9.91	E1	10	2.05	20.93
E2	7	6.43	181.00	E2	24	4.37	43.15	E2	5	3.89	38.93	E2	5	5.00	225.42
E3	17	4.50	20.51	E3	4	7.45	197.74	E3	3	5.32	48.40	E3	14	3.32	22.31
E4	22	3.41	117.33	E4	12	6.83	53.72	E4	4	4.16	15.20	E4	6	3.80	88.60
E5	23	2.85	20.14	E5	4	10.36	71.34	–	–	–	–	E5	5	7.24	30.63
E6	4	7.96	119.41	E6	7	4.82	137.81	–	–	–	–	E6	10	4.81	24.40
E7	8	6.74	33.39	E7	8	2.11	117.69	–	–	–	–	–	–	–	–

Where: N + _*Ri*_ - number of companies with positive *Ri*.

σ_(C)_ - Risk per Clustered Portfolio.

*Ri*_(C)_ - Clustered Portfolio Return.

### 4.3 Asset allocation results

After k-means clustering, select the companies per cluster to include in the portfolio.

Rules for asset allocation: In each different Technical Investment Strategy.

1. The maximum allowable number of companies in a portfolio was set to 10 (2 ≤ N ≤ 10)
2. If there are more than 10 companies who have positive *Ri* (Return), select 10 companies with the maximum return (*Ri*) per cluster.
3. “N/A” means less than 2 companies

Table 4, Table 5 shows that asset allocation for 2018 and 2020 data, respectively.

**Table 4:** Asset allocation for 2018 data.

MACD (12,26,9)			MACD (4,22,3)			MACD-ALMA (12,26,9)			MACD-ALMA (4,22,3)		
Cluster	N + *Ri*	AA	Cluster	N + *Ri*	AA	Cluster	N + *Ri*	AA	Cluster	N + *Ri*	AA
E1	40	10	E1	8	8	E1	3	3	E1*	1	N/A
E2	15	10	E2	17	10	E2	2	2	E2	4	4
E3	36	10	E3	25	10	–	–	–	E3	3	3
E4	20	10	E4	9	9	–	–	–	E4	8	8
E5	12	10	E5	26	10	–	–	–	–	–	–
E6	5	5	E6	22	10	–	–	–	–	–	–
E7	9	9	E7	11	10	–	–	–	–	–	–
E8	17	10	E8	49	10	–	–	–	–	–	–

**Table 5:** Asset allocation for 2020 data.

MACD (12,26,9)			MACD (4,22,3)			MACD-ALMA (12,26,9)			MACD-ALMA (4,22,3)		
Cluster	N + _*Ri*_	AA	Cluster	N + _*Ri*_	AA	Cluster	N + _*Ri*_	AA	Cluster	N + _*Ri*_	AA
E1	5	5	E1	23	10	E1	4	4	E1	10	10
E2	7	7	E2	24	10	E2	5	5	E2	5	5
E3	17	10	E3	4	4	E3	3	3	E3	14	10
E4	22	10	E4	12	10	E4	4	4	E4	6	6
E5	23	10	E5	4	4	–	–	–	E5	5	5
E6	4	4	E6	7	7	–	–	–	E6	10	10
E7	8	8	E7	8	8	–	–	–	–	–	–

Where: N + _*Ri*_ - number of companies with positive *Ri*, AA – asset allocation.

Note: *E1 of MACD-ALMA (4,22,3) should be removed since only 1 company in a cluster.

### 4.4 Portfolio optimization results

**Fig. 10:**
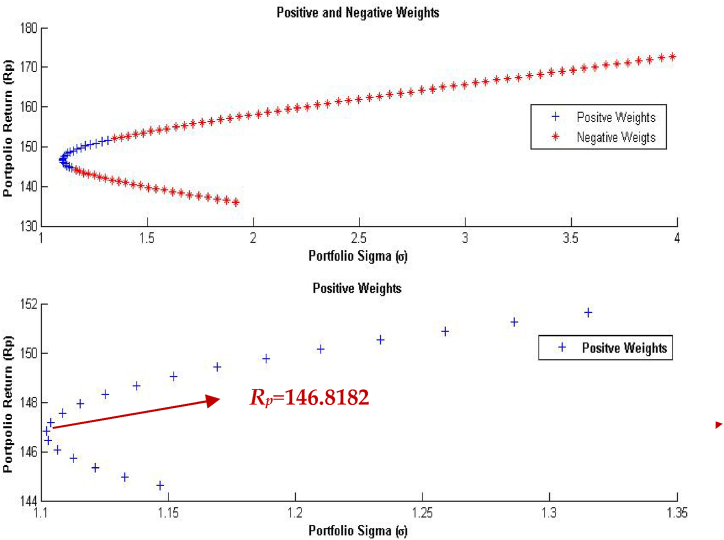
*R*_*P*_ and sigma of MACD (12, 26, 9), elbow method 4 (upper: Positive and negative weights; lower: Positive weights).

The mean-variance portfolio optimization model minimizes the risk (*σ*_*p*_) given a specified expected return (*R*_*P*_) in each portfolio. The optimization model will then suggest a weight for each asset/company on that portfolio. This paper also assumes that companies are independent of each other and therefore set the correlation parameter (ρ) to zero. Since *R*_*P*_ is subjectively determined by the investor, this paper examines different *R*_*P*_ that resulted in different minimum risk levels (*σ*_*p*_). The upper and lower limit of *R*_*P*_ used in this paper is the maximum and minimum values in a portfolio per cluster. The minimum risk (σp) between those ranges of *R*_*P*_ will result in *R*_*P*_ at a global minimum risk. Table 7, Table 9 show *R*_*P*_ (in %) at global minimum risk for 2018 and 2020 data, respectively. The resulting weights per portfolio was shown in Table 8, Table 10. Table 6 shows a Sample of portfolio optimization from MACD (12, 26, 9) of the Elbow Method, Cluster 4. Fig. 10 shows an *R*_*P*_ of 146.8182 and has a Global minimum risk of 1.102035 (See Table 6, Table 7). This method selects the minimum risk that generates positive weights and is called “*R*_*P*_ at global minimum risk” and negative weight has been eliminated in the simulations.

**Table 6:** Sample from MACD (12, 26, 9) of elbow 4.

*Rp*	*σ*_*p*_
144.5974	1.14671
144.9675	1.13286
145.3377	1.121485
145.7078	1.112663
146.0779	1.106455
146.4481	1.102903
**146.8182**	**1.102035**
147.1883	1.103856
147.5584	1.108353
147.9286	1.115494
148.2987	1.125229
148.6688	1.13749
149.039	1.152198
149.4091	1.16926
149.7792	1.188574
150.1494	1.210033
150.5195	1.233524
150.8896	1.258935
151.2597	1.286151
151.6299	1.31506

**Table 7:** Optimal portfolio for 2018 data.

MACD (12,26,9)			MACD (4,22,3)			MACD-ALMA (12,26,9)			MACD-ALMA (4,22,3)		
Cluster	*σ*_*p*_	*R*_*P*_	Cluster	*σ*_*p*_	*R*_*P*_	Cluster	*σ*_*p*_	*R*_*P*_	Cluster	*σ*_*p*_	*R*_*P*_
E1	0.48	116.11	E1	3.03	46.61	E1	0.86	2.83	E1*	N/A	N/A
E2	0.90	64.00	E2	1.61	110.66	E2	4.29	57.64	E2	1.76	5.88
E3	0.60	28.61	E3	1.05	74.03	–	–	–	E3	3.24	50.19
E4	1.10	146.82	E4	1.62	182.31	–	–	–	E4	0.55	4.11
E5	1.93	99.36	E5	0.47	107.49	–	–	–	–	–	–
E6	3.87	19.87	E6	0.65	153.22	–	–	–	–	–	–
E7	1.71	25.57	E7	1.33	23.34	–	–	–	–	–	–
E8	1.01	20.48	E8	0.59	41.68	–	–	–	–	–	–

**Table 8:** Optimal Portfolio weights for 2018 data.

Strategy	Cluster	*w*_*1*_	*w*_*2*_	*w*_*3*_	*w*_*4*_	*w*_*5*_	*w*_*6*_	*w*_*7*_	*w*_*8*_	*w*_*9*_	*w*_*10*_
MACD (12,26,9)	E1	0.0599	0.0900	0.0535	0.0854	0.0728	0.0656	0.2099	0.0750	0.2382	0.0498
	E2	0.0674	0.3255	0.2315	0.1020	0.0504	0.0493	0.0353	0.0464	0.0484	0.0439
	E3	0.0747	0.1155	0.1048	0.1129	0.0783	0.1212	0.0803	0.0598	0.1892	0.0632
	E4	0.0369	0.2049	0.2076	0.0750	0.0933	0.0462	0.0776	0.0436	0.0462	0.1689
	E5	0.0610	0.0829	0.0399	0.0867	0.1013	0.1747	0.0999	0.1782	0.1073	0.0680
	E6	0.1811	0.1937	0.2701	0.2438	0.1113	–	–	–	–	–
	E7	0.1338	0.1313	0.0709	0.1022	0.1198	0.0950	0.1334	0.1323	0.0813	–
	E8	0.1045	0.1153	0.0956	0.0899	0.0766	0.1066	0.0565	0.1263	0.0815	0.1471
MACD (4,22,3)	E1	0.0747	0.1571	0.1207	0.1492	0.1211	0.1477	0.1119	0.1177	–	–
	E2	0.1214	0.1585	0.0707	0.1001	0.1426	0.0729	0.0687	0.0836	0.0696	0.1118
	E3	0.0948	0.1109	0.0897	0.1485	0.1017	0.0691	0.1553	0.0892	0.0837	0.0571
	E4	0.0961	0.0777	0.1081	0.1697	0.1629	0.0649	0.0583	0.1617	0.1007	–
	E5	0.0988	0.0641	0.1827	0.0564	0.0831	0.0854	0.1083	0.1828	0.1006	0.0378
	E6	0.0736	0.0863	0.1120	0.0718	0.0961	0.1327	0.1377	0.0459	0.1398	0.1042
	E7	0.1116	0.0860	0.0839	0.0883	0.1546	0.1238	0.0693	0.1351	0.0431	0.1042
	E8	0.0889	0.0766	0.1571	0.0737	0.0761	0.1006	0.1414	0.0478	0.1776	0.0602
MACD-ALMA (12,26,9)	E1	0.1715	0.2633	0.5651	–	–	–	–	–	–	–
	E2	0.5700	0.4300	–	–	–	–	–	–	–	–
MACD-ALMA (4,22,3)	E2	0.2002	0.3217	0.2992	0.1789	–	–	–	–	–	–
	E3	0.4260	0.3266	0.2473	–	–	–	–	–	–	–
	E4	0.0928	0.0994	0.2425	0.1414	0.1080	0.0749	0.1092	0.1318	–	–

**Table 9:** Optimal portfolio for 2020 data.

MACD (12,26,9)			MACD (4,22,3)			MACD-ALMA (12,26,9)			MACD-ALMA (4,22,3)		
Cluster	*σ*_*p*_	*R*_*P*_	Cluster	*σ*_*p*_	*R*_*P*_	Cluster	*σ*_*p*_	*R*_*P*_	Cluster	*σ*_*p*_	*R*_*P*_
E1	4.46	48.19	E1	0.78	34.97	E1	0.90	9.36	E1	0.61	20.55
E2	2.36	180.23	E2	1.31	64.18	E2	1.69	39.30	E2	1.99	227.59
E3	1.34	30.38	E3	3.56	200.60	E3	3.04	50.87	E3	1.01	29.59
E4	0.92	130.64	E4	2.12	59.98	E4	1.99	16.19	E4	1.51	85.74
E5	0.82	29.53	E5	5.12	68.31	–	–	–	E5	3.22	28.51
E6	3.93	116.57	E6	1.77	136.15	–	–	–	E6	1.50	25.68
E7	2.35	32.97	E7	0.68	114.12	–	–	–	–	–	–

**Table 10:** Optimal Portfolio weights for 2020 data.

Strategy	Cluster	*w*_*1*_	*w*_*2*_	*w*_*3*_	*w*_*4*_	*w*_*5*_	*w*_*6*_	*w*_*7*_	*w*_*8*_	*w*_*9*_	*w*_*10*_
MACD (12,26,9)	E1	0.2287	0.2702	0.1409	0.1899	0.1703	–	–	–	–	–
	E2	0.1312	0.1939	0.1080	0.0872	0.1984	0.1648	0.1166	–	–	–
	E3	0.0959	0.0917	0.1153	0.1192	0.0916	0.1272	0.0670	0.1190	0.1106	0.0625
	E4	0.0740	0.1058	0.0538	0.0488	0.1627	0.0361	0.2081	0.0495	0.0505	0.2107
	E5	0.0999	0.0535	0.0700	0.0703	0.0549	0.1491	0.0858	0.1513	0.0669	0.1983
	E6	0.1783	0.2759	0.2590	0.2868	–	–	–	–	–	–
	E7	0.1471	0.0984	0.0856	0.1407	0.1195	0.1494	0.1275	0.1319	–	–
MACD (4,22,3)	E1	0.1387	0.1224	0.1199	0.0636	0.0703	0.1339	0.0687	0.1841	0.0558	0.0425
	E2	0.1250	0.0892	0.1130	0.0661	0.0955	0.1232	0.1209	0.0885	0.0782	0.1003
	E3	0.1704	0.4166	0.1996	0.2134	–	–	–	–	–	–
	E4	0.0997	0.0801	0.0832	0.0723	0.1350	0.1450	0.0929	0.0976	0.1103	0.0838
	E5	0.1842	0.2515	0.2959	0.2684	–	–	–	–	–	–
	E6	0.1104	0.1075	0.1840	0.1586	0.1544	0.1983	0.0867	–	–	–
	E7	0.0526	0.1114	0.1124	0.1336	0.1870	0.0679	0.2670	0.0682	–	–
MACD-ALMA (12,26,9)	E1	0.2663	0.1698	0.1002	0.4637	–	–	–	–	–	–
	E2	0.1647	0.3239	0.1640	0.1444	0.2030	–	–	–	–	–
	E3	0.4105	0.3219	0.2676	–	–	–	–	–	–	–
	E4	0.3450	0.2299	0.1374	0.2877	–	–	–	–	–	–
MACD-ALMA (4,22,3)	E1	0.0764	0.0901	0.1202	0.1716	0.0676	0.1465	0.0626	0.0550	0.0581	0.1517
	E2	0.4424	0.1068	0.1150	0.1148	0.2210	–	–	–	–	–
	E3	0.0866	0.1289	0.0728	0.1154	0.0904	0.0739	0.1279	0.1086	0.0654	0.1299
	E4	0.1335	0.1080	0.2237	0.2262	0.1625	0.1462	–	–	–	–
	E5	0.1787	0.1929	0.1949	0.1851	0.2483	–	–	–	–	–
	E6	0.0996	0.1149	0.1196	0.1249	0.1023	0.0791	0.0882	0.1146	0.0782	0.0786

Based on Table 7 (2018 data), *R*_*P*_ at global minimum risk ranges are 19.87%–146.82%, 23.34%–182.31%, 2.83%–57.64%, and 4.11%–50.19% for MACD (12, 26, 9), MACD (4,22,3), MACD-ALMA (12, 26, 9), and MACD-ALMA (4,22,3), respectively. From Table 9 (2020 data), the ranges are 29.53%–180.23%, 34.97%–200.60%, 9.36%–50.87%, and 20.55%–227.59%, respectively. For the MACD strategy, the result shows that the E4 portfolio using MACD (4,22,3) has a maximum *R*_*P*_ of 182.31% for the 2018 data while the E2 portfolio using MACD-ALMA (4,22,3) has a maximum *R*_*P*_ of 227.59% for the 2020 data. This also shows that the MACD-ALMA strategy managed a high-risk market condition but can achieve maximum return.

### 4.5 Validation of results using the next Year's historical price

To determine the performance of the TAKMV method, it was validated by applying the results and comparing them to the next year's historical price. In this case, 2018 results were compared to 2019 data, and 2020 results were compared to 2021 data. The comparison is applied to the same company per portfolio (simulation results and next year's data) for consistency. No validation was conducted between 2019 and 2020 since this was the transition of pre- and during-COVID-19 conditions.

**Fig. 11:**
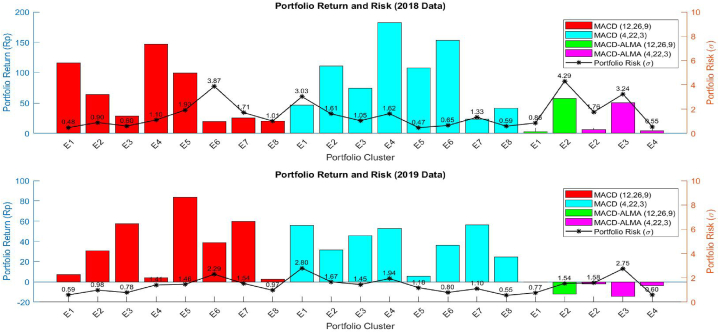
Portfolio risk and return results comparison per clustered portfolio (upper: 2018 data; lower: 2019 data).

**Fig. 12:**
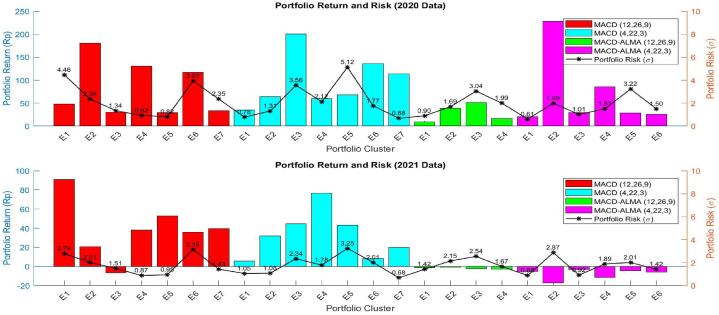
Portfolio risk and return results comparison per clustered portfolio (upper: 2020 data; lower: 2021 data).

Table 11, Table 12 and Fig. 11, Fig. 12 show the comparison of *R*_*P*_ applying the same weights for 2019 (2018 wt) and 2021 (2020 wt). %R_A_ and % R_B_ are the ratios of portfolio return for 2018–2019 and 2021–2022, respectively.

**Table 11:** Validation of 2018 Results to 2019 Data (Pre-Covid condition).

MACD (12,26,9)				MACD (4,22,3)				MACD-ALMA (12,26,9)				MACD-ALMA (4,22,3)			
Cluster	*R*_*P*__*(2018)*_	*R*_*P*__*(2019)*_	*%RA*	Cluster	*R*_*P*__*(2018)*_	*R*_*P*__*(2019)*_	*%RA*	Cluster	*R*_*P*__*(2018)*_	*R*_*P*__*(2019)*_	*%RA*	Cluster	*R*_*P*__*(2018)*_	*R*_*P*__*(2019)*_	*%RA*
E1	116.11	7.04	6.06%	E1	46.61	55.65	119.42%	E1	2.83	−0.32	−11.25%	E1*	N/A	N/A	
E2	64.00	30.82	48.17%	E2	110.66	31.63	28.59%	E2	57.64	−12.04	−20.89%	E2	5.88	−2.22	−37.67%
E3	28.61	57.46	200.83%	E3	74.03	45.70	61.73%	–	–	–		E3	50.19	−14.46	−28.81%
E4	146.82	4.04	2.75%	E4	182.31	52.55	28.83%	–	–	–		E4	4.11	−3.54	−86.19%
E5	99.36	83.46	84.00%	E5	107.49	5.52	5.14%	–	–	–		–	–	–	
E6	19.87	38.67	194.61%	E6	153.22	36.32	23.71%	–	–	–		–	–	–	
E7	25.57	59.54	232.89%	E7	23.34	56.23	240.97%	–	–	–		–	–	–	
E8	20.48	2.39	11.69%	E8	41.68	24.74	59.36%	–	–	–		–	–	–	

*%R*_*A*_ = (*R*_*P(2019)*_*/R*_*P(2018)*_)*100%.

**Table 12:** Validation of 2020 Results to 2021 Data (Covid condition).

MACD (12,26,9)				MACD (4,22,3)				MACD-ALMA (12,26,9)				MACD-ALMA (4,22,3)			
Cluster	*R*_*P*__*(2018)*_	*R*_*P*__*(2019)*_	*%RA*	Cluster	*R*_*P*__*(2018)*_	*R*_*P*__*(2019)*_	*%RA*	Cluster	*R*_*P*__*(2018)*_	*R*_*P*__*(2019)*_	*%RA*	Cluster	*R*_*P*__*(2018)*_	*R*_*P*__*(2019)*_	*%RA*
E1	48.19	90.85	188.50%	E1	34.97	5.69	16.27%	E1	9.36	−1.46	−15.61%	E1	20.55	−5.01	−24.40%
E2	180.23	20.65	11.46%	E2	64.18	31.85	49.62%	E2	39.30	−0.80	−2.04%	E2	227.59	−17.12	−7.52%
E3	30.38	−6.80	−22.37%	E3	200.60	44.64	22.25%	E3	50.87	−2.55	−5.02%	E3	29.59	−3.02	−10.22%
E4	130.64	38.09	29.16%	E4	59.98	76.84	128.12%	E4	16.19	−2.84	−17.54%	E4	85.74	−11.22	−13.09%
E5	29.53	52.57	178.03%	E5	68.31	43.22	63.28%	–	–	–		E5	28.51	−4.51	−15.83%
E6	116.57	35.68	30.61%	E6	136.15	8.21	6.03%	–	–	–		E6	25.68	−5.86	−22.80%
E7	32.97	39.62	120.20%	E7	114.12	19.78	17.33%	–	–	–		–	–	–	

%R_B_=(R_P(2021)_/R_P(2020)_)*100%.

Based on Table 11, Table 12, the results show that the MACD strategy, both (12, 26, 9) and (4,22,3) is effective in comparison to the MACD-ALMA strategy in terms of next year's portfolio return (*R*_*P*_). It is noticeable that MACD-ALMA resulted in negative *R*_*P*_. For 2018 results (pre-Covid-19), the maximum *%R*_*A*_ is 232.89% (E7) and 240.97% (E7) for MACD (12, 26, 9) and MACD (4,22,3), respectively, while for 2020 results (during-COVID-19), the maximum *%R*_*B*_ is 188.50% (E1) and 128.12% (E4) for MACD (12, 26, 9) and MACD (4,22,3), respectively.

## 5 Conclusions

The COVID-19 pandemic has been a worldwide health crisis. The Philippine stock market was seriously impacted by the global pandemic COVID-19. Retail investors continue to seek great ones in the damaged market. This paper uses three important analyses namely Technical Analysis, K-means Clustering, and Mean-Variance Portfolio Optimization Model (referred to as TAKMV Methodology) to determine possible portfolio and optimized portfolio returns while minimizing the expected risk. The optimal number of clusters was determined using the Elbow Method with average annual risk and an annual rate of return as an attribute.

A total of 230 assets/companies and 239 assets/companies for 2018 (pre-COVID-19 condition) and 2020 (during-COVID-19 condition), respectively, were used as a dataset. MACD and hybrid strategy (MACD-ALMA) with different windows were compared and analyzed to determine their performance during normal and COVID-19 conditions.

The application of technical analysis, clustering techniques, and portfolio optimization provides an efficient way to determine a pool of portfolios based on asset risk and return. Clustering and portfolio optimization will help to manage the portfolio selection and was based on the investor's risk criteria.

The technical analysis result shows that MACD works efficiently in the pre-COVID-19 conditions while MACD-ALMA works efficiently during-COVID-19 conditions, regardless of the number of assets with a positive annual rate of return. Using the Elbow Method for K-means clustering shows that the optimal number of clusters for 2018 and 2020 data are different. Portfolio optimization result shows that optimal return can be obtained using the MACD strategy in a pre-COVID-19 condition and MACD-ALMA during-COVID-19 condition. This also shows that the MACD-ALMA strategy manages a high-risk market condition and can achieve maximum return during COVID-19 conditions.

To determine the performance of the TAKMV method, it was validated by applying its results and comparing it to the next year's historical price. The 2018 results were compared to 2019 data and the 2020 results were compared to 2021 data. The comparison is applied to the same company per portfolio for consistency. No validation was conducted between 2019 and 2020 since this was the transition of pre- and during-COVID-19 conditions. Validation of results using next year's historical price shows that the MACD strategy is more effective compared to MACD-ALMA.

Overall, Technical Analysis can help traders and investors to identify trends and patterns in the Philippine stock market during the pandemic. With the pandemic causing significant economic disruption, technical analysis can help traders to identify potential buying and selling opportunities based on market trends and patterns. While K-means clustering can be used to create a diversified portfolio of stocks that can help reduce risk during volatile market conditions. Additionally, the Mean-Variance Model can be used to develop investment strategies that maximize returns while minimizing risk. Which can be used to allocate funds across different sectors of the Philippine stock market based on market trends and risk factors.

With the use of the TAKMV method, investors, students, and decision-makers can make more profitable investment decisions in the Philippine stock market using Technical Analysis, Machine Learning, and Mean-Variance Model.

### 5.1 Theoretical contribution

This study provides theoretical contributions to the existing literature by using the three important analyses (Technical Analysis, K-Means Clustering, and Mean-Variance Portfolio Optimization Model) named TAKMV methodology to determine possible portfolio and optimized portfolio returns while minimizing the expected risk amidst the COVID-19 pandemic. First, it enhanced stock selection: The combination of Technical Analysis, K-means clustering, and Mean-Variance model can provide investors with a more comprehensive approach to stock selection. This can lead to more informed investment decisions and potentially higher returns. Second, it improved risk management: The Mean-Variance model can help investors to manage risk by selecting portfolios that optimize returns for a given level of risk. The use of K-means clustering and Technical Analysis can further enhance risk management by identifying stocks that are less correlated with each other and potentially less risky. Third, Technical Analysis can help investors to identify market trends and potential turning points. The use of K-means clustering can provide a more detailed analysis of market trends by grouping stocks with similar price movements. Fourth, it also improved portfolio diversification. The use of K-means clustering and the mean-variance model can help investors to create diversified portfolios that are less exposed to specific industries or stocks. This can reduce the risk of significant losses due to market or industry-specific events. And lastly, it identifies new investment opportunities. The use of K-means clustering, and Technical Analysis can help investors to identify new investment opportunities that may have been overlooked by traditional analysis methods.

The integration of Technical Analysis, K-means clustering, and Mean-Variance model can provide a more comprehensive approach to stock selection and risk management in the Philippine stock market during the COVID-19 pandemic. The theoretical contributions of this approach include enhanced stock selection, improved risk management, a better understanding of market trends, improved portfolio diversification, and identification of new investment opportunities. These contributions can help investors to make more informed investment decisions, potentially leading to higher returns and reduced risk.

### 5.2 Practical implication

This paper has implications for practitioners, decision-makers, and managers. Using the combined methods of technical analysis, K-means clustering, and mean-variance portfolio optimization model, this paper develops a cluster trading strategy that allows for building a diversified portfolio with technical analysis. This paper provides a step-by-step procedure for assessing, clustering, selecting, and optimizing portfolios while considering the risk and its return. This method can be used by engineers, managers, institutional, and retail investors to select the best stock portfolios; they will gain all the benefits of diversification in this study as this will help to protect an investor's portfolio from the systematic risk that could expose the portfolio to losses. In addition, broad market pullbacks such as the 2020 pandemic situation obviously can suffocate the entire portfolio without any good strategy employed.

The proposed TAKMV Method will reduce the extreme downside losses in any market condition. Importantly, this method can be applied to other financial markets such as foreign exchange and cryptocurrency exchange, which are popular and trending among retail investors.

### 5.3 Limitations and future research

This paper limits the assessment using previous data to test and validate the performance of the proposed TAKMV method. Return estimation with the use of forecasting tools and techniques can be considered in future research such as Holt-Winters, neural networks, or other forecasting techniques, and can be employed together with other evolutionary optimization methods. Also, the application of TAKMV to other financial markets such as foreign exchange and cryptocurrency exchange as well as applications of it to post-pandemic conditions. Lastly, other technical investment strategies can be explored and utilized in the TAKMV method.

## Author contributions

Conceptualization, M.M.N., and M.N·Y.; methodology, M.M.N., M.N.Y. and J.V.T; validation, M.M.N., M.N·Y., and J.V.T; formal analysis, M.M.N., M.N.Y. and J.V.T.; investigation, M.M.N, and M.N·Y.; resources, M.M.N., M.N.Y. and J.V.T.; data curation, M.M.N., and M.N·Y.; writing—original draft preparation, M.M.N., M.N.Y. and J.V.T.; writing—review and editing, M.N·Y., Y.T.P. and J.V.T.; visualization, M.N.Y. and Y.T.P.; supervision, M.N·Y., Y.T.P. and J.V.T.; project administration, M.M.N., M.N·Y., and J.V.T.; funding acquisition, M.N·Y., and Y.T.P. All authors have read and agreed to the published version of the manuscript.

## Funding

This research was funded by Mapúa University Directed Research for Innovation and Value Enhancement (DRIVE).

## Author contribution statement

Maricar M. Navarro: Conceived and designed the experiments; Performed the experiments; Analyzed and interpreted the data; Contributed reagents, materials, analysis tools or data; Wrote the paper.

Michael Nayat Young: Conceived and designed the experiments, Analyzed and interpreted the data, Wrote the paper.

Yogi Tri Prasetyo: Analyzed and interpreted the data; Wrote the paper.

Jonathan V. Taylar: Analyzed and interpreted the data; Wrote the paper.

## Data availability statement

Data will be made available on request.

## Declaration of competing interest

The authors declare that they have no known competing financial interests or personal relationships that could have appeared to influence the work reported in this paper.

## References

[1] Gherghina Ș.C.a., Joldeș C.C. (2020). Stock market reactions to COVID-19 pandemic outbreak: quantitative evidence from ARDL bounds tests and granger causality analysis. Int. J. Environ. Res. Publ. Health.

[2] Elsayed A., Abdelrhim M. (2020).

[3] Al-Awadhi A.M., Alsaifi K., Ahmad A., Alhammadi S. (2020). Death and contagious infectious diseases: impact of the COVID-19 virus on stock market return. J. Behav. Experimen. Finan..

[4] Kartal M.T., Depren S.K., Depren Ö. (2021). How main stock exchange indices react to covid-19 pandemic: daily evidence from east asian countries. Global Econ. Rev..

[5] Xu D. (2022). Canadian stock market volatility under COVID-19. Int. Rev. Econ. Finance.

[6] Yiu M.S., Tsang A. (2021). Impact of COVID-19 on ASEAN5 stock markets. Journal of Asia Pacific.

[7] Ozkan O. (2021).

[8] Hatmanu M., Cautisanu C. (2021). The impact of COVID-19 pandemic on stock market: evidence from Romania. Int. J. Environ. Res. Publ. Health.

[9] Kartal M.T., Ertuğrul H.M., Ulussever T. (2022). The impacts of foreign portfolio flows and monetary policy responses on stock markets by considering COVID-19 pandemic: evidence from Turkey. Borsa Istanbul Review.

[10] Li B., Hoi S.C., Sahoo D., Liu Z.-Y. (2015). Moving average reversion strategy for on-line portfolio selection. Artif. Intell..

[11] Li T., Zhang W., Xu W. (2015). A fuzzy portfolio selection model with background risk. Appl. Math. Comput..

[12] Markowitz H. (1952). Portfolio selection. J. Finance.

[13] Sui Y., Hu J., Ma F. (2020). A possibilistic portfolio model with fuzzy liquidity constraint. Complexity.

[14] Li B., Hoi S.C. (2014). Online portfolio selection: a survey. ACM Comput. Surv..

[15] Chang K.H., Young M.N. (2019). Behavioral stock portfolio optimization considering holding periods of B-stocks with short-selling. Comput. Oper. Res..

[16] Moreno D., Petrakisab M. (2022). The impact on market outcomes of the portfolio selection of large equity investors. Econ. Lett..

[17] Rodríguez Y.E., Gómez J.M., Contreras J. (2021). Diversified behavioral portfolio as an alternative to modern portfolio theory. N. Am. J. Econ. Finance.

[18] Harris R.D.F., Mazibas M. (2022). Portfolio optimization with behavioural preferences and investor memory. Eur. J. Oper. Res..

[19] Bi J., Jin H., Meng Q. (2018). Behavioral mean-variance portfolio selection. Eur. J. Oper. Res..

[20] Ayala J., Torres M.G., Noguera J.L.V., Vela F.G., Divina F. (2021). Technical analysis strategy optimization using a machine learning approach in stock market indices. Knowl. Base Syst..

[21] Hafner C.M., Wang L. (2022). Dynamic Portfolio Selection with Sector-specific Regularization. Econom. Statist..

[22] Katsikis V.N., Mourtas S.D., Stanimirović P.S., Li S., Cao X. (2022). Time-varying mean–variance portfolio selection problem solving via LVI-PDNN. Comput. Oper. Res..

[23] Ghahtarani A. (2021). A new portfolio selection problem in bubble condition under uncertainty: application of Z-number theory and fuzzy neural network. Expert Syst. Appl..

[24] Zhang Y., Lin H., Yang X., Long W. (2021). Combining expert weights for online portfolio selection based on the gradient descent algorithm. Knowl. Base Syst..

[25] Gong X., Yu C., Min L. (2021). A cloud theory-based multi-objective portfolio selection model with variable risk appetite. Expert Syst. Appl..

[26] Ahmadi E., Jasemi M., Monplaisir L., Nabavi M.A., Mahmoodi A., Jam P.A. (2018). New efficient hybrid candlestick technical analysis model for stock market timing on the basis of the support vector machine and heuristic algorithms of imperialist competition and genetic. Expert Syst. Appl..

[27] Chiang W.C., Enke D., Wu T., Wang R. (2016). An adaptive stock index trading decision support system. Expert Syst. Appl..

[28] Weng B., Ahmed M.A., Megahed F.M. (2017). Stock market one-day ahead movement prediction using disparate data sources. Expert Syst. Appl..

[29] Alhashel B.S., Almudhaf F.W., Hansz J.A. (2018). Can technical analysis generate superior returns in securitized property markets? Evidence from east asia markets. Pac. Basin Finance J..

[30] Henrique B.M., Sobreiro V.A., Kimura H. (2018). Stock price prediction using support vector regression on daily and up to the minute prices. J. Finan. Data Sci..

[31] Patel J., Shah S., Thakkar P., Kotecha K. (2015). Predicting stock market index using fusion of machine learning techniques. Expert Syst. Appl..

[32] Nakano M., Takahashi A., Takahashi S. (2018). Bitcoin technical trading with artificial neural network. Phys. Stat. Mech. Appl..

[33] Petrusheva N., Jordanoski I. (2016). Comparative analysis between the fundamental and technical analysis of stocks. J. Process Manag. New Technol..

[34] Tsai C.F., Hsiao Y.C. (2010). Combining multiple feature selection methods for stock prediction: union, intersection, and multi-intersection approaches. Decis. Support Syst..

[35] Sedighi M., Jahangirnia H., Gharakhani M., Farahani Fard S. (2019). A novel hybrid model for stock price forecasting based on metaheuristics and support vector machine. Data.

[36] Picasso A., Merello S., Ma Y., Oneto L., Cambria E. (2019). Technical analysis and sentiment embeddings for market trend prediction. Expert Syst. Appl..

[37] Ozkok F.O., Celik M. (2021). A hybrid validity index to determine K parameter value of k-means algorithm for time series clustering. Int. J. Inf. Technol. Decis. Making.

[38] Gubu L., Rosadi D., Abdurakhman (2021). A new approach for robust mean-variance portfolio selection using trimmed k-means clustering. Indust. Engin. Manag. Syst..

[39] Alwarid A., Sihabuddin A. (2022). An absolute differences K-means clustering approach on determining intervals to optimize fuzzy time series M arkov Chain model. Int. J. Intellig. Engin. Syst..

[40] Fang Z., Chiao C. (2021). Research on prediction and recommendation of financial stocks based on K-means clustering algorithm optimization. J. Comput. Methods Sci. Eng..

[41] Seong N., Nam K. (2021). Predicting stock movements based on financial news with segmentation. Expert Syst. Appl..

[42] Gavira-Durón N., Gutierrez-Vargas O., Cruz-Aké S. (2021). Markov chain K-means cluster models and their use for companies' credit quality and default probability estimation. Mathematics.

[43] Bukhsuren E., Namsrai O.E. (2021). Methodology in securities portfolio selection from the stock exchange listed companies. Smart Innov. Syst. Technol..

[44] Muangprathub J., Intarasit A., Boongasame L., Phaphoom N. (2020). Portfolio risk and return with a new simple moving average of price change ratio. Wireless Pers. Commun..

[45] Marketwatch. https://www.marketwatch.com.

[46] Appel G. (1979).

[47] Sanel H. (2016). MACD analysis of weaknesses of the most powerful technical analysis tool. Indepen. J. Manag. Prod..

[48] Kang B.K. (2021). Improving MACD technical analysis by optimizing parameters and modifying trading rules: evidence from the Japanese nikkei 225 futures market. J. Risk Financ. Manag..

[49] Aguirre A.A.A., Medina R.A.R., Méndez N.D.D. (2020). Machine learning applied in the stock market through the Moving Average Convergence Divergence (MACD) indicator. Invest. Manag. Financ. Innovat..

[50] Tan P.-N., Steinbach M., Karpatne A., Kumar V. (2019).

[51] Govindarajulu U., Bedi S. (2022). K-means for shared frailty models. BMC Med. Res. Methodol..

[52] Sayli A., Akbulut C., Kosuta K. (2018). Multiple regression analysis system in machine learning and estimating effects of data Transformation&Min-max normalization. J. Engin. Technol. Applied Sci..

[53] Omar T., Zohdy M., Rrushi J. (2021). 2021 IEEE International Conference on Consumer Electronics (ICCE).

[54] Kizys R., Doering J., Juan A.A., Polat O., Calvet L., Panadero J. (2022). A simheuristic algorithm for the portfolio optimization problem with random returns and noisy covariances. Comput. Oper. Res..

