# Corrigendum to “Stock market optimization amidst the COVID-19 pandemic: Technical analysis, K-means algorithm, and mean-variance model (TAKMV) approach” [9(7) (July 2023) e17577]

**DOI:** 10.1016/j.heliyon.2023.e18640

**Published:** 2023-08-03

**Authors:** Maricar M. Navarro, Michael Nayat Young, Yogi Tri Prasetyo, Jonathan V. Taylar

**Affiliations:** a School of Industrial Engineering and Engineering Management, Mapúa University, 658 Muralla St., Intramuros, Manila, 1002, Philippines; b School of Graduate Studies, Mapúa University, 658 Muralla St., Intramuros, Manila, 1002, Philippines; c Department of Industrial Engineering, Technological Institute of the Philippines Quezon City, 938 Aurora Blvd, Cubao, Quezon City, 1109, Metro Manila, Philippines; d International Bachelor Program in Engineering, Yuan Ze University, 135 Yuan-Tung Road, Chung-Li, 32003, Taiwan; e Department of Industrial Engineering and Management, Yuan Ze University, 135 Yuan-Tung Road, Chung-Li, 32003, Taiwan; f Department of Computer Engineering, Technological Institute of the Philippines Quezon City, 938 Aurora Blvd, Cubao, Quezon City, 1109, Metro Manila, Philippines

In the original published version of this article, the author has uploaded incorrect Fig. 1 as shown below.

**Fig. 1:**
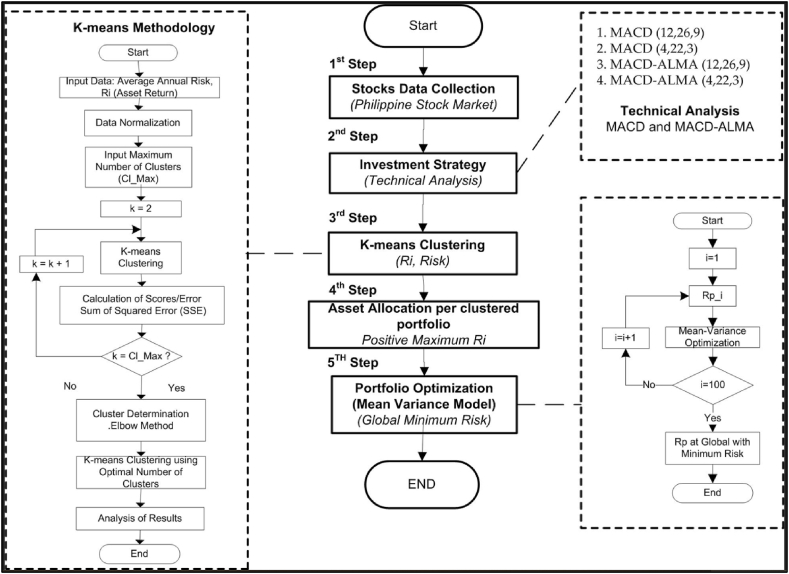
Flowchart of the proposed TAKMV methodology.

